# Mechanisms of Interleukin-10-Mediated Immunosuppression in Viral Infections

**DOI:** 10.3390/pathogens14100989

**Published:** 2025-10-01

**Authors:** Zijing Guo, Qifu He, Yan Zhang, Yuling Li, Zhidong Zhang

**Affiliations:** 1 Key Laboratory of Veterinary Medicine in Universities of Sichuan Province, College of Animal Science and Veterinary Medicine, Southwest Minzu University, Chengdu 610041, China; zijingguo7@163.com (Z.G.); yanzhang6782@163.com (Y.Z.); yulingl25487@163.com (Y.L.); 2 Wuhou District Health Hospital for Women & Children, Chengdu 610041, China; 18080046224@163.com

**Keywords:** interleukin-10, immunosuppression, viral infections, innate and adaptive immunity, therapeutic intervention

## Abstract

Interleukin-10 (IL-10), a potent anti-inflammatory cytokine, plays a vital role in regulating immune responses across various infectious and inflammatory conditions. While IL-10 is essential for preventing excessive tissue damage and maintaining immune homeostasis (e.g., respiratory syncytial virus), its elevated levels could result in immunosuppression during viral infections, enabling viruses to evade host defenses (e.g., foot-and-mouth disease virus). This review aims to elucidate the mechanisms through which IL-10 mediates immunosuppression in viral infections and to explore the implications of these mechanisms for therapeutic intervention. The key scientific concepts outlined in this review include the mechanisms of IL-10 production and its varied impacts on the immune response during viral infections. Specifically, we discuss the multifaceted inhibitory effects of IL-10 on innate and adaptive immunity, including its implications for antigen presentation, T cells activation, pro-inflammatory cytokine production, immune cell differentiation, trafficking, apoptosis, and co-inhibitory expression related to T cells exhaustion. Finally, we discuss the therapeutic potential of targeting IL-10, such as monoclonal antibodies and small molecule inhibitors, and their potential to restore effective immune responses. By summarizing current knowledge on IL-10’s role in viral infections, this review offers a thorough insight into its immunosuppressive mechanisms and their therapeutic potential, paving the way for innovative treatment strategies in viral diseases.

## 1. Introduction

The immune system constitutes a sophisticated system of organs, tissues, and cells that work together to protect the body against pathogens. Among the myriads of molecules that regulate immune responses, cytokines occupy a key position in modulating the duration and intensity of these reactions. Interleukin-10 (IL-10) is a key cytokine with a profound impact on modulating immune responses, particularly during viral infections [1,2]. IL-10 could suppress the production of pro-inflammatory cytokines (e.g., TNF-α, IL-6), thereby limiting inflammation and tissue damage during acute viral infection (e.g., SARS-CoV-2 [3], H1N1 [4], respiratory syncytial virus (RSV) [5]). Conversely, IL-10 can inhibit immune cell activation and function, thereby facilitating viral persistence and contributing to chronic infections (e.g., human immunodeficiency virus (HIV), Hepatitis C virus (HCV), lymphocytic choriomeningitis virus (LCMV), human papillomavirus (HPV)) [6,7] and in certain acute viral infections (e.g., dengue virus [8] and foot-and-mouth disease virus (FMDV) [9]). Identify predictive biomarkers indicative of this immunological balance that could aid in disease diagnosis and treatment.

Immunosuppression is characterized by a reduced or absent ability to generate an effective response, which predisposes individuals to secondary infections and co-infections with other pathogens. This compromised immune function exacerbates the severity of the disease and greatly increases the fatality rate [10,11]. Moreover, immunosuppression adversely affects the immunogenicity of other pathogenic vaccines, potentially leading to immune failure [12,13]. Preventing and controlling infectious diseases in humans or animals remains challenging, causing substantial economic damage to breeding sectors worldwide. Numerous studies have elucidated the mechanisms of IL-10-mediated immunosuppression, such as inhibiting T cells activation and function, inhibiting antigen-presenting cells (APCs) function, and modulating T cells differentiation [3,9,14]. The precise mechanisms by which IL-10 causes immunosuppression during viral infections remain still not fully understood due to various challenges, such as (1) limitations of in vivo models (e.g., compensatory upregulation of TGF-β/IL-35 in IL-10R^-/-^ mice) [15]; (2) viral strategy heterogeneity (e.g., PRRSV structural proteins activating IL-10 via TLR-MAPK p38 vs. cytomegalovirus-encoded IL-10 (cmvIL-10) [16,17]; and (3) cell-type-specific responses (e.g., Macrophage-specific (but not T-cell) IL-10 receptor signaling was crucial for anti-TNF-induced CD206+ regulatory macrophage differentiation and therapeutic efficacy) [18]. This review aims to recapitulate the cellular sources of IL-10, the mechanisms of its production, and the molecular pathways involved in viral infection-driven immunosuppression. This review offers valuable perspectives on the role of IL-10 in mediating immunosuppression caused by viral infections, thereby identifying potential therapeutic targets for addressing IL-10-mediated immunosuppressive effects in viral pathogenesis.

## 2. Methods

**Figure 1:**
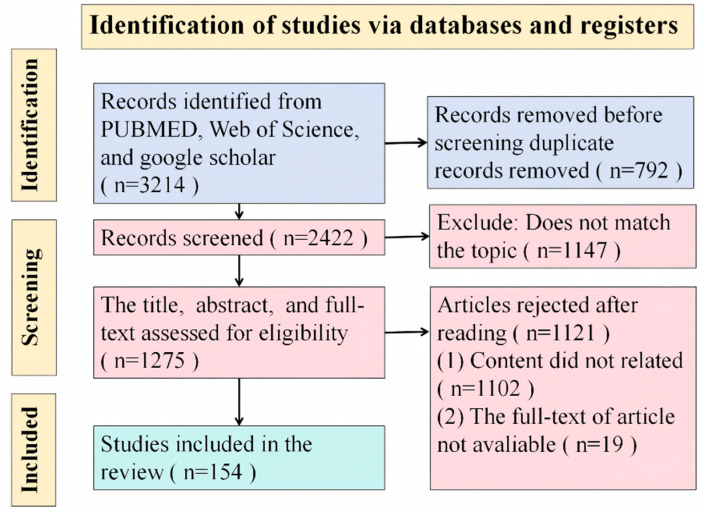
The flow diagram of search strategies, data screening, exclusion, and inclusion of the studies in the review.

The comprehensive data search was carried out with PUBMED (https://www.ncbi.nlm.nih.gov/pubmed/, accessed on 23 January 2025), Web of Science (https://webofscience.clarivate.cn/wos/woscc/smart-search, accessed on 23 January 2025), and Google Scholar (https://scholar.google.com/, accessed on 23 January 2025) databases. The keywords used to the datasets in each search engine included “IL-10 and immunosuppression”, “interleukin-10 and immunosuppression”, “interleukin-10 and viral infection”, “IL-10 and viral infection”, “interleukin-10 and virus infection”,“IL-10 and virus infection”, “interleukin-10 and therapeutic”, “IL-10 and therapeutic”, “IL-10 and innate immunity”, “IL-10 and adaptive immunity”, “IL-10 Family”, “IL-10 structure”, “IL-10 structure”, “IL-10 production”, “IL-10 and antigen presentation”, “IL-10 and T cells activation”, “IL-10 and cell differentiation”, “IL-10 and immune cell apoptosis”, “IL-10 and co-inhibitory molecular”, “IL-10 and T cells exhaustion”. Literature Screening Criteria: Inclusion Criteria: The empirical research papers directly relate to the research topic. They have a complete methodology description. Exclusion Criteria: conference abstracts, non-peer-reviewed literature, and duplicated research screening process. The PRISMA flowchart was used to present the three-stage quality assessment of the initial screening, full-text screening, and final inclusion. The overview of the studies included or excluded in the review is provided in a flow chart (Figure 1).

## 3. IL-10 Production During Viral Infections

### 3.1. The IL-10 Family of Cytokines

IL-10, as a homodimeric protein, consists of two polypeptide chains, each comprising 160 amino acids, organized in a compact form to form a soluble 36 kDa homodimer [19]. IL-10 dimer exhibits 3D domain swapping, with the C-terminus of one monomer positioned adjacent to the N-terminus of its partner through antiparallel association [20]. This spatial arrangement inspired the engineering of a covalently stabilized dimer by connecting these termini. Its V-shaped configuration is maintained by two disulfide bonds within the chain and characterized by six α-helical structures [20]. This distinctive architecture facilitates the binding or engagement of IL-10 with its receptor complex comprising IL-10Rα and IL-10Rβ. IL-10Rα is a private receptor subunit that engages IL-10 with high affinity, and IL-10Rβ is a shared receptor subunit that engages IL-10 with extremely low affinity [21]. A recent study found that the hexameric IL-10 receptor complex exhibits a stelliform structure, where an all α-helical IL-10 homodimer bridges two IL-10Rα and IL-10Rβ subunits via CRH domains, forming three interaction sites [22]. Site 1 involves high-affinity polar/electrostatic contacts between IL-10Rα and IL-10 helices α1/α5, while site 2 features IL-10Rβ loops L2/L3 clasping IL-10 helix α3 via 15 hydrogen bonds and 9 salt bridges, with key hydrophobic interactions mediated by Tyr82 and Tyr59. Site 3 comprises stem contacts between receptor subunits. IL-10Rα primarily serves to recruit IL-10 to the cell surface; it is the extent of engagement of IL-10Rβ that dictates the subsequent extent of intracellular STAT3 activation. Mutational studies confirm critical roles of Asp25/Glu96 in IL-10 for electrostatic interactions with IL-10Rβ, governing STAT3 activation [22].

IL-10 is an important part of the IL-10 cytokine family, which also comprises IL-19, IL-20, IL-22, IL-24, IL-26, IFN-λ1 (IL-29), IFN-λ2 (IL-28A) and IFN-λ3 (IL-28B) [23]. The family is subdivided into three subgroups based on genomic organization, structural motifs, and receptor utilization. The first subgroup includes only IL-10, which binds the receptor complex comprising IL-10R1 and IL-10R2, initiating a signaling cascade that modulates various functions of immune cells, including its dual role in limiting immunopathology while facilitating immunosuppression and persistent infection [4]. The second subgroup, designated the IL-20 subfamily (IL-19, IL-20, IL-22, IL-24, IL-26), primarily orchestrates epithelial barrier defense against bacterial and fungal pathogens [23]. These cytokines engage combinatorial receptor complexes: IL-19, IL-20, and IL-24 signal through IL-20R1/IL-20R2, whereas IL-20 and IL-24 additionally utilize IL-22R/IL-20R2. The third subgroup consists of type III interferons (IFN-λs: IL-28A, IL-28B, IL-29), which share IL-10R2 as a β-chain but couple with IL-28Rα to form antiviral receptor complexes [23]. An intriguing aspect of IL-10 biology involves virally encoded homologues (vIL-10s), such as cytomegalovirus-encoded IL-10 (cmvIL-10) and latency-associated cytomegalovirus-encoded IL-10 (LAcmvIL-10), which mimic human IL-10’s immunosuppressive functions through structural mimicry of the cellular cytokine [2]. These viral homologues selectively bind IL-10R1 with high affinity but fail to activate full anti-inflammatory signaling, thereby subverting host immunity by dampening Th1 responses and promoting viral persistence [2]. Despite functional diversification, all subgroups exhibit evolutionary ties to immune evasion strategies, with vIL-10s representing a paradigm of pathogen-driven cytokine co-option.

### 3.2. The Role of IL-10 During Viral Infections

IL-10 is a highly pleiotropic cytokine; it can elicit significant anti-inflammatory and pro-inflammatory effects. IL-10 preserves immune balance by restraining excessive immune activation, primarily by preventing the production of inflammatory mediators [1]. In contrast, IL-10 paradoxically exerts an inflammatory effect on activated CD8+ T cells, enhancing production of the pro-inflammatory cytokine interferon (IFN)-γ [24]. Additionally, the potent immunosuppressive activity exerted by IL-10 remains a central research focus, encompassing inhibition in antigen-presenting machinery, attenuation of T lymphocyte activation, and induction of immune cell death, etc. [1,14]. The elevated IL-10 level represents a notable feature following numerous viral infections, such as SARS-CoV-2 [25], influenza A virus [26], LCMV [6], HIV [27], porcine reproductive and respiratory syndrome virus (PRRSV) [28], porcine circovirus type 2 (PCV2) [29,30], FMDV [31], and others (Table 1). In infection with RSV, IL-10 can mitigate excessive inflammation and prevent severe immunopathology. Specifically, IL-10 influences the adaptive immune response by controlling T cell-driven inflammation and pulmonary tissue damage [32]. Conversely, the elevated IL-10 level is correlated to viral persistence and disease progression after HIV, HCV, and HBV infections [2]. Additionally, the host’s production of IL-10 results in widespread immunosuppression via multiple mechanisms. FMDV induces immunosuppression through IL-10-mediated lymphopenia and T cell dysfunction [9,31]. Similarly, IL-10, known for its potent suppressive effects on monocyte differentiation, is associated with increased susceptibility to infection with PRRSV by enhancing the expression of CD163 and CD169 in monocytes [28]. IL-10 exhibits distinct biological functions depending on the stage of infection, whether acute or chronic. During acute SARS-CoV-2 infection, IL-10 inhibits T cell expansion; however, following viral clearance, IL-10 encourages lung effector T cells to differentiate into CD69+ CD103+ tissue-resident memory cells [3]. Overall, IL-10, as a pivotal immunoregulatory cytokine, exerts dualistic effects on the host immune response. The biological outcomes of IL-10 signaling are context-dependent, being modulated by multiple variables including: (1) the viral pathogens (e.g., DNA or RNA viruses), (2) temporal dynamics of infection (acute or chronic phases), (3) cellular sources of IL-10 production (immune or non-immune cells), and (4) target cell populations receiving IL-10 signals.

**Table 1:** The role of IL-10 during some viral infections in humans or other mammals.

Virus	The Main Role in the Immune Response	States	Cellular Origin	Induce Viral Protein	Reference
SARS-CoV-2	(1). Inhibition of T cell expansion (2). Enhancement of the differentiation of lung effector T cells into CD69+CD103+ tissue resident memory cells (3). Anti-viral activity and anti-inflammatory effects (4). Enhancement of ACE2 receptor mRNA expression in lung-derived and endothelial cells (5). Impairment of the MAIT cell response (6). Disease severity	Acute infection	Unknown	Unknown	[3,32,33,34,35]
H1N1	(1). Anti-inflammatory (2). Prediction of progression to a fatal outcome in H1N1	Acute infection	Unknown	Unknown	[36]
RSV	(1). Disease severity (2). Decrease in IFN-I secretion by alveolar macrophages (3). Inhibition of disease and inflammation (4). Dampening of effector T cell responses (5). Inhibition of pro-inflammatory cytokines and chemokines	Acute infection	CD4+ T cells, CD8+ T cells, nBreg cells	Unknown	[5,32,37,38]
HIV	(1). Increased of CD16high monocytes, sCD163 and sCD14 (2). Inhibition of NK cell functions (3). The development of comorbidities in patients with HIV (4). Inhibition of adaptive immune responses and inflammation	Chronic infection	Bregs, NK cells, CD8+ T cells, monocyte	Tat protein, gp41 envelope protein	[39,40,41,42,43]
SIV	(1). Establishment of the reservoir and persistence (2). Inhibition of the inflammation response (3). Loss of CD4+ T cells (4). Increase in virus replication	Chronic infection	B cells (B10 cells)	Unknown	[7,44]
HAV	Impact on the immune response and liver damage	Acute infection	Unknown	Unknown	[45]
HBV	(1). Increased severity of chronic HBV infection (2). Prediction of the prognosis of patients with (3). Inhibition of effector T-cells (4). Enhancement of regulatory T-cells (5). Inhibition of cytotoxic CD4+ T cell activity	Chronic infection, acute-on-chronic liver failure	Bregs, B Cells	Unknown	[46,47,48]
HCV	(1). Increase in susceptibility to chronic HCV (2). Predictive marker of recovery from an active HCV infection (3). Inhibition of CD4+ and CD8+ T cells	Chronic infection	B cells, CD4+ primary T cells	HCV-core protein	[49,50].
HEV	Unknown	Acute infection	γδ cells	Unknown	[51]
LCMV	(1). Functional exhaustion of antiviral CD8+ and CD4+ T cells (2). Decrease in cytokine production (3). Inhibition of the proliferative potential of NK cells (4). Enhancement of virus replication	Chronic infection	Dendritic cells, Macrophage, T cell, NK cells	Unknown	[52,53]
CSFV	Unknown	Unknown	PK-15 cells, monocyte-derived dendritic cells	Erns, E1, and E2	[54,55]
FMDV	(1). Lymphopenia is involved in downregulating apoptosis, trafficking, and the coinhibitory expression of lymphocytes (2). Inhibition of T cell proliferation	Acute infection	Macrophage	Unknown	[9,31,56]
ASFV	Interfering with immune responses by controlling antiviral IFN levels and a cell-mediated immune response	Unknown	Unknown	Unknown	[57]
PRRSV	(1). Immunosuppression (2). Enhancement of virus replication via enhancing CD169 expression	Unknown	MoDC, porcine alveolar macrophages	N protein, nsp2, and nsp5	[12,17,28,58,59,60]
PCV2	(1). Promotion of PCV2 persistent infection by aggravating the tissue lesions through suppression of T cell infiltration (2). The thymic depletion of pigs	Unknown	Macrophages	Rep, Cap	[29,61,62,63,64]
TMEV	Decrease in the kinetics of virus clearance at early times after infection and ameliorating disease at later times	Unknown	T cells	Unknown	[65]
HPV	(1). Immunosuppression (2). Enhancement of virus persistence		Cervical cancer cells	HPV E2, E6 and E7 proteins	[66,67]

### 3.3. Cellular Sources and the Regulation of IL-10 Production

The cellular sources of IL-10 are diverse, encompassing both immune and non-immune cells. Among immune cells, key producers of IL-10 include T cells, such as CD4+ T cells, in particular Foxp3+ Treg, CD8+ T cells, alongside B cells, macrophages, dendritic cells (DCs), natural killer (NK) cells [68], regulatory innate lymphoid cells (ILCs) [69], neutrophils, mast T cells, myeloid-derived suppressor cells (MDSCs) [70], and eosinophils. Among these, T cells, DCs, macrophages, and MDSCs are identified as the main cellular sources. IL-10 can also be produced by non-immune cells, such as epithelial cells, keratinocytes, and some types of tumor cells, when certain conditions are met. The secretion of IL-10 is tightly controlled by multiple signaling pathways, which can change depending on the cell type, the viral pathogens (e.g., DNA or RNA viruses), and environmental conditions [71].

#### 3.3.1. T Cells

**Figure 2:**
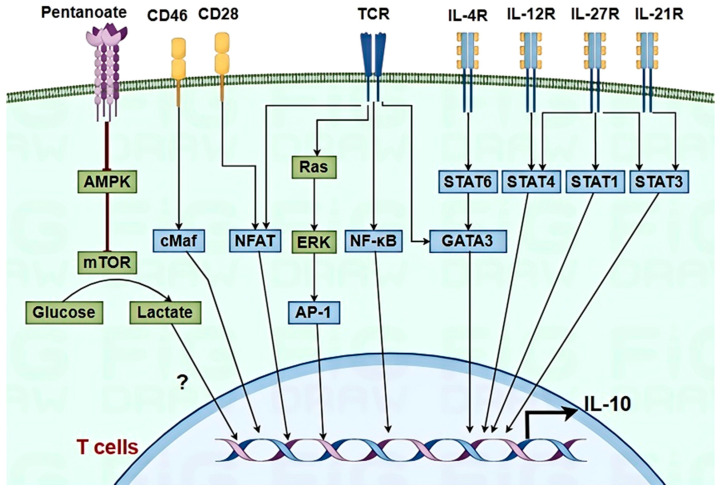
Signaling pathways regulating IL-10 expression in T cells. “?” indicates that the regulatory mechanism is still unclear. In T cells, the activation of the TCR and co-stimulatory molecules, such as CD28 and CD46, initiates the synthesis of IL-10 through a complex signaling cascade involving NF-κB, NFAT, and c-Maf. This process is often accompanied by the secretion of cytokines, including IFN-γ, IL-4, IL-27, and IL-21, which activate specific STAT proteins—STAT4, STAT6, and STAT3. In the nucleus, transcription factors such as AP-1, GATA3, and RORγt enhance IL-10 transcription. Metabolic reprogramming, like short-chain fatty acids pentanoate, modulates IL-10 production.

In T cells, IL-10 production is governed by a complex signaling network (Figure 2). Activation of the T cell receptor (TCR) and co-stimulatory molecules such as CD28 and CD46 can trigger IL-10 synthesis through pathways involving nuclear factor-kappa B (NF-κB), c-Maf, mitogen-activated protein kinase (MAPK), and nuclear factor of activated T cells (NFAT), thereby enhancing IL-10 transcription [1,72,73]. IL-10 expression in CD4+ T cells is often accompanied by the release of hallmark cytokines such as IFN-γ, IL-4, IL-27, and IL-21, and the receptors for these cytokines can activate signal transducer and activator of transcription (STAT) proteins that are involved in IL-10 expression, including STAT4, STAT6, and STAT3, in the cytoplasm of Th1, Th2, and Th17 T cells, respectively [1,73]. Within the nucleus, transcription factors interact with the IL-10 promoter, inducing the expression of various transcription factors, including AP-1, GATA binding protein 3 (GATA3), retinoid-related orphan receptor γt (RORγt), Blimp-1, c-Maf, and Bhlhe40, etc. [74,75]. Additionally, metabolic reprogramming in the T cells was involved in IL-10 production [1]. Extracellular ATP, hypoxia, oxysterols, or cholesterol biosynthesis inhibition in T regulatory type 1 (Tr1) and Th1 cells modulate IL-10 expression [76]. Pentanoate, a short-chain fatty acid, is believed to prevent the activation of AMPK, thereby releasing the mechanistic target of the rapamycin (mTOR) pathway, increasing glycolytic flux, and facilitating IL-10 secretion in Th17 cells [77]. Viral infection of T cells driving glycolytic reprogramming remains unsubstantiated due to viral tropism constraints (e.g., FMDV) and technical hurdles in T cell isolation. In individuals with SARS-CoV-2 infection, T cells exhibit pronounced activation states and glycolysis metabolic characteristics driven by an in vivo manifestation [78]. Under virus infection conditions, the signal pathway of IL-10 production in T cells remains unclear.

#### 3.3.2. Macrophages and DCs

**Figure 3:**
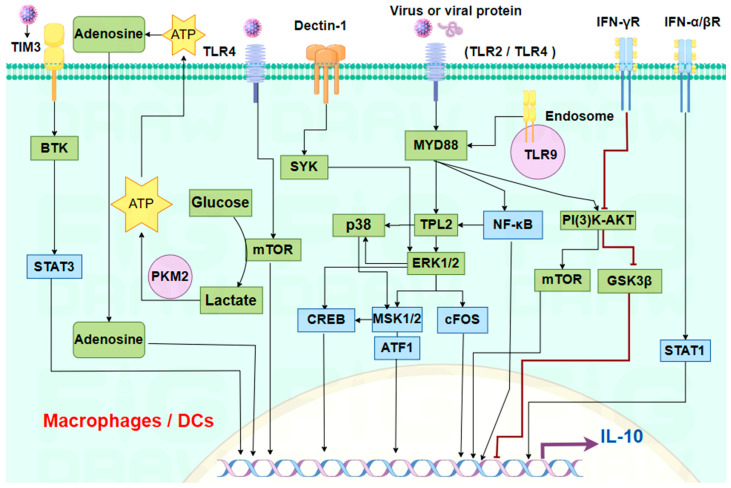
Signaling pathways regulating IL-10 expression in macrophages and DCs. In macrophages and mDCs, the expression of IL-10 is primarily mediated by TLRs, particularly TLR2, TLR4, and TLR9. Upon TLR engagement, signaling cascades are activated via MYD88, which triggers downstream MAPK and NF-κB pathways. MAPKs, including ERK1/2 and p38, regulate IL-10 expression through transcription factors, such as CREB, MSK1/2, and c-Fos. The PI3K/Akt pathway also modulates IL-10 expression. Metabolic reprogramming towards glycolysis influences IL-10 production in response to LPS stimulation. TIM-3 can enhance IL-10 expression in DCs via the TIM-3-BTK-STAT3 signaling axis.

In macrophages and DCs, the production of IL-10 is primarily regulated via Toll-like receptors (TLRs) (specifically TLR2, TLR4, and TLR9) (Figure 3). After TLR ligand engagement, signaling pathways are triggered via the Toll/IL-1 receptor (TIR) domain, like myeloid differentiation primary-response protein 88 (MYD88) [71]. TLRs can induce IL-10 expression via MAPK, NF-κB, and phosphoinositide-3-kinase (PI3K) serine/threonine protein kinase B (Akt) pathways [1,14,79]. MAPKs are critical in regulating IL-10, and MAP3 kinase tumor progression locus 2 (Tpl2) can activate downstream ERK1/2 and P38 pathways. This activation results in the production of cyclic adenosine monophosphate (cAMP) response element-binding protein (CREB), cAMP-dependent transcription factor 1 (ATF1), c-Fos, and Sp1, ultimately regulating IL-10 expression [1]. The direct binding of NF-κB to the IL-10 promoter can induce the expression of IL-10. Finally, the PI3K/Akt cascade modulates the generation of IL-10 via ERK1/2 and mTOR activation, and the PI3K/AKT inhibits IL-10 production by inhibiting the kinase glycogen synthase kinase 3β (GSK3β) [80]. During viral infection, PCV2 directly infects porcine alveolar macrophages, which may lead to IL-10 production through the MyD88-NF-κB pathway [30]. Furthermore, the Rep protein of PCV2 also induces the expression of IL-10 through the p38-MAPK pathway in macrophages [29]. Previous studies showed that PRRSV also activates IL-10 production through NF-κB and p38 MAPK pathways in porcine alveolar macrophages [17]. Beyond direct pattern recognition receptor (PRR) signaling, co-stimulatory receptors can influence the generation of IL-10 [81,82]. Previous studies indicated that FMDV not only induces high levels of IL-10 but also enhances the expression of PD-1, TIM-2, TIGIT, and CTLA-4 [9]. T cell immunoglobulin and mucin-3 (TIM-3), as co-suppressor molecules, can activate expression of IL-10 in DCs through the TIM-3-BTK-STAT3 signaling axis [82]. Metabolic reprogramming in immune cells, particularly the shift towards glycolysis in macrophages and DCs, further influences IL-10 expression. A recent study suggested that the glycolytic enzyme pyruvate kinase M2 (PKM2) causes the production of IL-10 via adenosine derived from glycolytic ATP [83] (Figure 3). It was noted that FMDV activates glycolysis and hijacks HK2 to inhibit innate immunity [84]. Whether FMDV can induce the production of IL-10 through the glycolytic pathway remains to be further investigated. It is worth studying the intricate regulatory mechanisms of IL-10 production in specific cell types.

## 4. Mechanisms of IL-10-Mediated Immunosuppression

**Figure 4:**
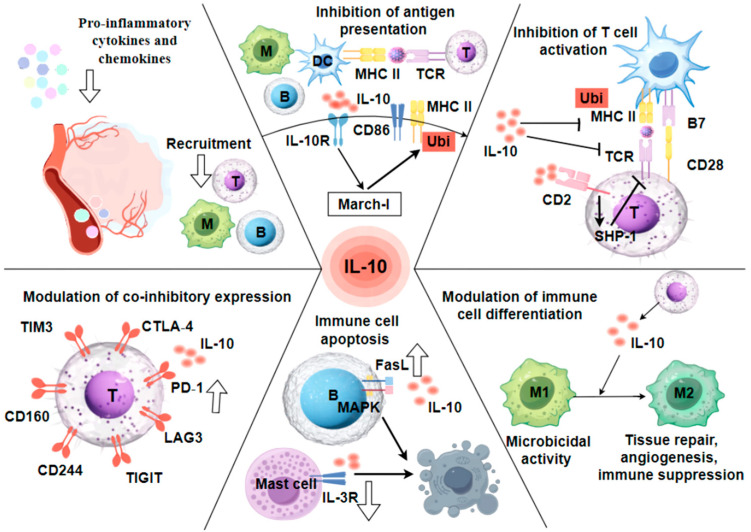
The mechanisms of IL-10-mediated immunosuppression. IL-10 exerts multifaceted inhibitory effects by suppression of pro-inflammatory cytokine secretion; blockade of TLR-mediated dendritic cell activation; downregulation of APC surface co-stimulatory molecules and MHC-II complexes; impairment of macrophage differentiation; induction of programmed cell death pathways; and promotion of T cell exhaustion states.

IL-10 exerts pleiotropic suppressive effects on innate and adaptive immune responses, encompassing inhibition of recruitment and activation of immune cells, impairment of antigen-presenting machinery, attenuation of T lymphocyte activation, regulation of immune cell differentiation trajectories, induction of apoptotic pathways, and upregulation of co-inhibitory receptors implicated in T cell exhaustion phenotypes, etc. (Figure 4). Understanding the mechanisms by which IL-10 contributes to immunosuppression contributes to developing effective antiviral strategies and therapeutic interventions.

### 4.1. Reduction in Recruitment and Activation of Immune Cells

In the context of viral infections, IL-10 exerts anti-inflammatory via reducing the production of pro-inflammatory cytokines and chemokines, including TNF-α, IL-1β, IL-6, and IL-12, etc. [85]. This suppression of inflammatory mediators can lead to reduced recruitment and activation of T cells, B cells, NK cells, and neutrophils, which are not beneficial for early viral control [86]. During Orf virus (ORFV) infection, it encoded IL-10 homologues (ORFV-IL-10) that limited the recruitment of blood-derived monocytes, dendritic cells, and mature mast cells into inflamed skin [87]. ORFV-IL-10 also suppressed the activation of dendritic cells within the skin, reducing their trafficking to the draining lymph node [87]. Moreover, IL-10 also suppresses the synthesis of IFN-γ, a potent antiviral cytokine. In chronic viral infections, the sustained IL-10 production can lead to decreased IFN-γ production from virus-specific T cells, thereby contributing to viral persistence [88]. A recent study revealed that IL-10 selectively inhibited IRF5 and IRF1 activity (not NF-κB/MAPK pathways), leading to downregulation of inflammatory genes and interferon responses. Epigenetic mechanisms included reduced chromatin accessibility and H3K27ac marks at IRF1-associated enhancers [89].

**Figure 5:**
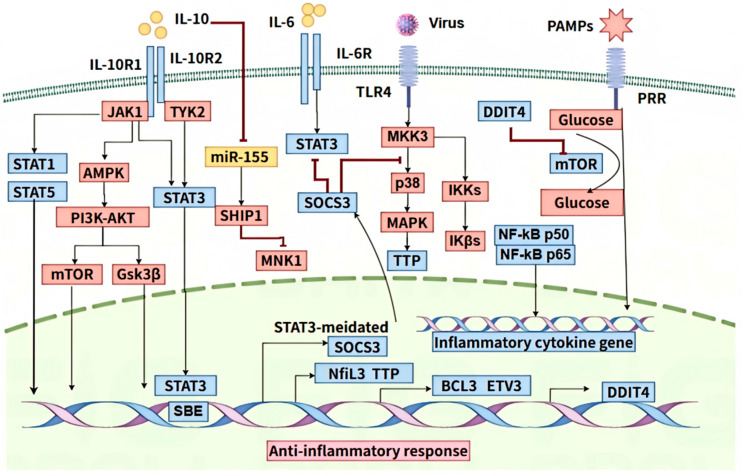
Mechanisms by which IL-10 suppresses pro-inflammatory cytokine production. IL-10 mediates its anti-inflammatory effects through engagement with its receptor complex, comprising IL-10R1 and IL-10R2. IL-10 binds to IL-10R, activating the JAK1-TYK2-STAT3 signaling pathway, which orchestrates the transcription of various anti-inflammatory mediators. Key transcription factors ETV3 and Nfil3 are activated, leading to the suppression of NF-κB activity and a reduction in IL-12p40 production. Furthermore, STAT3 modulates the activity of MAPK p38 and RNA-binding proteins like TTP, resulting in decreased levels of pro-inflammatory cytokines. The IL-10/STAT3 axis also plays a role in chromatin remodeling, indicating an epigenetic layer of regulation. In addition to the JAK/STAT3 pathway, the PI3K/Akt/GSK3β signaling cascade contributes to IL-10-mediated transcriptional regulation in macrophages. Other downstream mediators, including Bcl3 and A20-binding inhibitors, require further investigation to elucidate their contributions to the comprehensive anti-inflammatory effects of IL-10.

The binding of IL-10 to its receptor IL-10R activates the JAK1-TYK2-STAT3 signaling cascade, ultimately leading to STAT3-mediated anti-inflammatory effects [90]. STAT3 activates a cascade of anti-inflammatory mediators, including transcription factors like ETV3 and Nfil3, which suppress NF-κB-mediated transcription and IL-12p40 production, respectively [91,92] (Figure 5). Additionally, STAT3 modulates the activity of MAPK p38 and RNA destabilizing factors like tristetraprolin (TTP), leading to decreased levels of inflammatory cytokines [93,94]. Furthermore, STAT3-dependent IL-10 inhibits miR-155 transcription, upregulating SHIP1, a negative regulator of TLR signaling and TNF translation [95]. Recent findings that chromatin accessibility modulated by STAT3 targets like HDAC4 suggest an epigenetic regulation by the IL-10-STAT3 axis [96].

While STAT3 exhibits a vital effect in IL-10 mediating the anti-inflammatory response, the STAT3 pathway may not account for the full spectrum of IL-10’s actions. Other mediators downstream of IL-10R, such as Bcl3 and A20-binding inhibitors, remain to be fully elucidated in terms of their STAT3 dependence. In a STAT3-dependent way, IL-10 modulates the metabolic regulator DDIT4, a regulator of mTOR, IL-10R signaling restricts glycolytic flux following TLR4 activation, thereby regulating the macrophage’s inflammatory response (Figure 5). Beyond the JAK/STAT3 pathway, accumulating evidence identifies the PI3K/Akt/GSK3β cascade as a parallel signaling pathway mediating IL-10-induced transcriptional regulation in macrophages [97,98] (Figure 5). This pathway demonstrates particular therapeutic potential through its dual regulatory capacity: (i) GSK3β-dependent selective suppression of LPS-induced IL-1β, IL-8, and COX-2 (while preserving TNF-α and SOCS3 expression) [4], and (ii) AMPKα1-mediated coordination with mTORC1 to direct macrophage polarization [5]. While STAT1 and STAT5 have been documented as additional signaling effectors downstream of IL-10 receptor activation [99]—particularly in FcγRI and CD14 regulation [10]—their pleiotropic effects pose challenges for targeted therapeutic intervention [99] (Figure 5). In contrast, the PI3K/Akt axis exhibits superior pathway specificity through its differential modulation of discrete inflammatory mediators. A recent study demonstrated that IL-10 exhibits anti-inflammatory effects in macrophages by upregulating enzymes responsible for fatty acid desaturation and influencing the downstream regulation of the NF-κB family transcription factor REL [100]. This complexity underscores the multifaceted nature of IL-10’s anti-inflammatory mechanisms, involving intricate gene regulatory networks orchestrated by STAT3 while permitting context-dependent engagement of alternative pathways.

### 4.2. Inhibition of Antigen Presentation

Antigen presentation is a critical process within the immune system that facilitates the recognition and elimination of viruses. In this process, immunoproteasomes break down the virus, and the resulting antigenic peptides are presented on major histocompatibility complex (MHC) molecules. These MHC-peptide complexes are subsequently transported to the APC’s surface to interact with TCR on T cells. While all nucleated cells can function as APCs, only specific subsets, known as professional APCs, specialize in activating and proliferating antigen-specific T cells [101]. Professional APCs express both MHC class I and MHC class II, including DCs, monocytes, macrophages, and B cells. In contrast, non-professional APCs primarily express MHC class I [102,103]. IL-10 plays a crucial role in suppressing antigen presentation, a key process in initiating and maintaining effective antiviral action [14]. A recent study showed that human cytomegalovirus-IE2 protein inhibits the antigen presentation function of macrophages through the IL-10/STAT3 signaling pathway [104].

**Figure 6:**
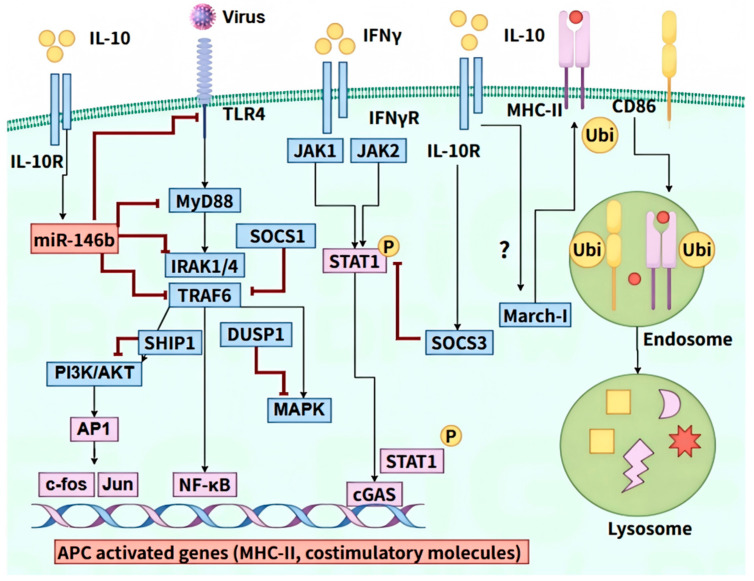
Mechanisms of IL-10 in suppressing antigen presentation. “?” indicates that the regulatory mechanism is still unclear. IL-10 attenuates TLR-dependent activation of APCs by inhibiting TLR signaling through multiple mechanisms. IL-10 signaling promotes the STAT3-dependent expression of miR-146b, SOCS1, SHIP1, and DUSP1, which collectively disrupt TLR signaling, with miR-146b specifically targeting TLR4, MyD88, IRAK1/4, and TRAF6. IL-10 also influences IFNγ-mediated activation of macrophages and monocytes, leading to the production of SOCS3 that inhibits JAK2-dependent STAT1 activation. Furthermore, IL-10 modulates APCs’ function post-activation, downregulating MHC-II expression in monocytes via March-I, while leaving mature dendritic cells unaffected in activation status.

Firstly, IL-10 impacts TLR-dependent APCs activation by dampening TLR signaling through multiple pathways, including miRNA-mediated decline of TLR signaling components, ubiquitin-induced breakdown of TLR signaling components, and direct inhibition of MyD88-dependent and MyD88-independent TLR signaling [14]. IL-10 signaling activates STAT3, which then transcribes miR146b, SOCS1/3, SHIP1, DUSP1, and AMP kinase, all of which disrupt TLR signaling. Specifically, miR-146b inhibits the expression of TLR4, MyD88, IRAK1/4, and TRAF6 proteins, whereas SOCS1, DUSP1, and SHIP1 inhibit the activation of IRAK1/4, p38, and PI3K, respectively [105,106] (Figure 6). A previous study showed that HBV stimulation on intrahepatic myeloid-derived cells (iMDCs) could also induce IL-10 production in a TLR2-dependent way; IL-10 blockade could partially abolish iMDC-suppressed T cell activation [107].

Secondly, IL-10 affects IFNγ-dependent activation of mononuclear cells/macrophages and antigen processing. Unlike DCs, macrophages and monocytes lack constitutive MHC-II expression, which emerges post-IFNγ activation, thereby enabling APCs to function via costimulatory molecules. When IFNγ binds to its receptor on monocytes/macrophages, it triggers the phosphorylation of STAT1, which then moves into the nucleus and binds to activated sequences on genes targeted by IFNγ. However, IL-10 signaling results in the production of SOCS3, further inhibiting the activation of STAT1 in response to IFNγ signaling [108] (Figure 6). Furthermore, IL-10 directly affects antigenic peptide processing and peptide-MHC molecule complex type II (pMHC-II) assembly. IL-10, either alone or in conjunction with IFN-γ, reduces the expression of cathepsin S in macrophages, thereby hindering pMHC-II complex formation [109]. Additionally, it also suppresses IFNγ-driven MHC-II transcription, enhancing its inhibitory effect [109].

Lastly, IL-10 modulates the function of APCs following their activation. While IL-10 exerts a suppressive role on the activation of APCs, it does not alter the activation status of mature DCs, as evidenced by unchanged expression levels of MHC-II, CD86, CD40, and pMHC-II presenting capacity [110]. In monocytes, IL-10 decreases MHC-II via March-I, an E3 ubiquitin ligase for degradation [111] (Figure 6). While March-I is induced by IL-10 in monocytes/macrophages, it is constitutively expressed in non-activated DCs and diminishes upon activation. Studies suggest March-I involvement in the inhibition of IL-10 in DC’s function. However, direct suppression of DC activation by IL-10 may also limit March-I’s physiological downregulation. Another study also indicated that IL-10 suppressed TLR-mediated DC activation independently of March-I, affecting APCs’ function [14]. Consequently, the role of March-I in DC activation warrants further investigation.

### 4.3. Inhibition of T Cells Activation and Expansion

As previously discussed, IL-10 suppresses the expression of co-stimulatory molecules and MHC-II on APCs, which are essential for effective T cells activation. This reduction in co-stimulatory signals further contributes to the overall suppression of T cells activity [14]. cmvIL-10 downregulates key co-stimulatory molecules (CD40, CD80, CD86, CD83) on MDDCs, impairing surface expression of these critical markers and thereby inhibiting efficient T cell activation [112]. Additionally, IL-10 directly reduces TCR signaling, thereby suppressing T cell activation. Research has demonstrated that IL-10 can interfere with CD2 or CD28-mediated co-stimulation via the activation of SHP-1, a phosphatase that negatively regulates TCR signaling [113]. By suppressing these early activation events, IL-10 can substantially weaken T cells responses to viral antigens [113,114]. During chronic infections with HCV, IL-10 inhibits the priming of naïve virus-specific CD8+ T cells, which brings about a reduced amount of virus-specific T cells and impaired differentiation of effector cells, which may facilitate viral persistence [115]. During acute infection with FMDV, IL-10 production by DCs is markedly elevated. Notably, the use of a neutralizing antibody against porcine IL-10 restored T cell activation mediated by DCs [31]. A recent study demonstrates that Coxsackievirus B3 (CVB3) infection triggers excessive IL-10 production by neutrophils. This elevated IL-10 enhances IL-10R1 expression on macrophages, activating the STAT3-IL-6/IL-10 signaling axis while suppressing IL-12 and MHC II expression. Consequently, these changes inhibit CD8+ T cell activation and compromise viral clearance [116]. These findings underscore the multifaceted role of IL-10 in modulating T cells activation, highlighting its significance in both chronic and acute viral infections.

### 4.4. Modulation of Immune Cell Differentiation

IL-10 has been demonstrated to induces functional reprogramming in macrophages, transitioning them from a classically activated (M1) phenotype—characterized by pro-inflammatory cytokine (TNF-α/IL-6/IL-12) production and microbicidal activity—to an alternatively activated (M2) phenotype produce IL-10/TGF-β and Arg1, related to tissue repair, angiogenesis, and immunosuppression [117]. Lentiviral IL-10 expression induces stable M2 macrophage reprogramming, mitigating inflammation in chronic disease models. The shift from M1 to M2 is essential for reducing inflammation and reestablishing tissue balance [118,119]. Mechanically, IL-10 primarily induces M2 polarization through the activation of the IL-10-IL-10R-STAT3 signaling pathway [120,121]. Additionally, IL-10 downregulated miR-155 and activated SOCS5, thereby promoting macrophage M2 polarization [117]. IL-10 can also induce M2 differentiation by regulating other cytokines (suppressing TNF-a/IL-6 while enabling IL-4 secretion [122]) and through feedback loops (particularly in the pathological macrophage polarization of polycystic kidney disease, where autocrine IL-10 sustains this self-reinforcing cycle [123]). A previous study showed that PRRSV modulates the switch of macrophage polarization from M1 to M2 by upregulating the MoDC-induced secretion of CD83, accompanied by an increase in the cytokine IL-10 [124]. In PRRSV infection, whether IL-10 is involved in the M2 polarization of macrophages and the underlying mechanism is worthy of further study.

Early-phase IL-10 also exerts cell-extrinsic immunosuppression during acute LCMV infection, attenuating effector Th1 responses and impairing memory CD4+/CD8+ T cell generation. IL-10 signaling blockade during priming enhances memory T cell functional competence, skews the Th1/Tfh differentiation balance by reducing virus-specific Tfh frequency. It is noted that IL-10 exhibits complex roles in CD8+ T cell memory differentiation. Previous studies indicate that IL-10 secreted by Treg cells can aid in the differentiation of CD8+ T cell memory by protecting some of these cells from inflammatory signals during acute LCMV infections [125], while others suggest IL-10 may impede memory development of CD8+ T cells under similar conditions or have no discernible effect following IL-10 blockade [126]. Persistent antigens drive CD8+ T cell differentiation into exhausted progenitor (Texprog) and terminally exhausted (Texterm) cells. STAT3—primarily activated by IL-10—is essential for effector CD8+ T cell differentiation during acute infection. It transcriptionally upregulates effector genes while suppressing Texprog-associated programs, and cooperates with BATF/IRF4 to remodel chromatin at effector loci [127]. These discrepancies, emerging from diverse experimental setups, underscore a delicately controlled system where CD8+ memory formation may be modulated by the intensity of T cells signal and IL-10’s spatial-temporal dynamics.

### 4.5. Induction of Immune Cell Apoptosis

The death of immune cells is also one of the ways that contributes to immunosuppression. There are various ways for cell death, including apoptosis, autophagy, necroptosis, pyroptosis, ferroptosis, cuproptosis, and inflammatory programmed cell death (PANoptosis), etc. However, IL-10 plays a certain role in cellular immune apoptosis. Previous studies have shown that IL-10 triggers apoptosis in lymphocytes, mast cells, and macrophages through an intrinsic pathway that encompasses the cleavage of Caspase-3 and the depolarization of mitochondria [128,129]. IL-10 can downregulate signaling molecules, such as Syk, Lyn, and Akt. Apoptosis inhibitors Bcl-xl and Bcl-2, can impact the normal function of mitochondria [128]. Additionally, IL-10 may induce mast cells apoptosis by reducing IL-3R expression and STAT5 phosphorylation [128]. Furthermore, rIL-10 can increase the expression of FasL on B-1a cells [130]. IL-10 generated by CD9+ regulatory B cells in patients with severe asthma has been shown to trigger T cell apoptosis through the MAPK-dependent pathway [131]. The elevated IL-10 in lupus can activate Fas and caspase, leading to lymphocyte apoptosis [132]. Our previous study has demonstrated that the increased IL-10 in vivo can induce the apoptosis of peripheral T cells in mice infected FMDV, and the apoptosis of isolated mouse T cells treated with rIL-10 can also be significantly induced [9]. A recent study showed that CmAb-(IL10)2, an IL-10 fusion protein derived from cetuximab, prevents dendritic cell-induced apoptosis of CD8+ tumor-infiltrating lymphocytes by controlling the production of IFN-γ [133]. However, IL-10 could prevent spontaneous apoptosis in monocytes via inducing Flip, a crucial inhibitor of the Fas-death signal. Moreover, the distinctive feature of IL-10 resides in its capacity to suppress the induction of B-cell apoptosis [134]. These findings underscore the dual role of IL-10 in modulating apoptosis within immune cells, highlighting its capacity to both induce and suppress apoptotic processes. The contrasting effects of IL-10 may be attributed to the specific contexts in which it acts on different immune cell populations, thereby influencing the overall immune response.

### 4.6. Co-Inhibitory Molecular Expression of T Cells Exhaustion

T cell exhaustion refers to a state of impaired T cell function that occurs in various chronic infections and malignancies [135]. T cell exhaustion is a gradual state marked by a growing weak of effector function, together with heightened presentation of various immune checkpoint inhibitors, including programmed cell death receptor 1 (PD-1), cytotoxic T lymphocyte antigen-4 (CTLA-4), T-cell immunoglobulin domain and mucin domain-containing protein 3 (TIM3), lymphocyte activation gene protein-3 (LAG3), T-cell immunoreceptor with Ig and ITIM domains (TIGIT), 2B4 (CD244), and CD160 [136]. Chronic infection with viruses such as HIV [137], LCMV [138], and HCV [139] can lead to T cell exhaustion. A recent study showed that T cell exhaustion was also observed during LCMV acute infection [140]. It is noted that these viral infections/co-infections induce T cells exhaustion, accompanied by a high level of IL-10 [141]. Our prior study demonstrated that the presentation of co-inhibitory molecules was increased following acute infection with FMDV, accompanied by elevated levels of IL-10. Moreover, the knockout of IL-10 and the blockade of IL-10/IL-10R signaling in vivo led to a downregulation of TIGIT and 2B4 expression on CD8+ T cells, as well as CTLA-4 expression on CD4+ T cells [9]. While IL-10 blockades reverse T cell function in specific chronic models (e.g., LCMV Clone 13), this effect is not universal. For highly virulent strains (e.g., LCMV Docile), IL-10 blockade fails to sustain viral clearance or prevent long-term exhaustion [142]. Thus, it is speculated that the reversal of T cell exhaustion markers following IL-10 blockade in chronic infections appears to be highly context-dependent and potentially transient.

Among these, PD-1 is acknowledged as the primary inhibitory controller of T cell activity. Its signaling pathway results in the downregulation of AKT, phosphoinositide 3-kinase, extracellular signal-regulated kinase, and phosphoinositide phospholipase C-γ, along with controlling the cell cycle [143]. Ultimately, it leads to reduced production of IFN-γ/IL-2, decreased cell proliferation, and increased apoptosis [143]. According to a previous report, IL-10 can trigger high expression of PD-L1 on peripheral blood monocyte cells through STAT3 [144]. In contrast, TIGIT is upregulated during chronic LCMV infection, and TIGIT modulates IL-10 production in vivo [145]. TIGIT restricts immune-mediated tissue damage during acute virus infection in an IL-10-dependent manner during acute LCMV and influenza virus infection [145]. These findings underscore the critical role of IL-10 in regulating co-inhibitory receptor expression, thereby influencing T cell exhaustion in chronic infections and malignancies

## 5. Therapeutic Implications and Future Directions

### 5.1. Utilizing IL-10 Modulation in Antiviral Therapies

IL-10 has strong anti-inflammatory and immunosuppressive effects, which affects viral infections in a dual manner. While it can mitigate excessive immune responses, thereby preventing tissue damage, it may also inadvertently promote viral persistence. Modulating IL-10 signaling—whether through inhibition or enhancement—can significantly alter disease progression by influencing immune responses and the rate of viral clearance [146]. IL-10 has been considered for conditions characterized by excessive inflammation, showing dual anti-fibrotic and anti-inflammatory effects in pulmonary fibrosis while alleviating viral-induced acute lung injury/acute respiratory distress syndrome (ARDS), positioning it as a potential coronavirus disease 2019 (COVID-19) and other therapeutic candidate [32]. Notably, a recent study showed that a potent platform via exosome-based systemic and repeated delivery of engineered IL-10 mRNA has been established in controlled inflammation control [147]. Differently, a study showed that IL-10 deficiency exacerbates RSV-induced weight loss and pulmonary dysfunction compared to controls [32]. Thus, rIL-10 treatments may help reduce severe inflammatory responses in diseases like RSV-induced pulmonary disease, potentially improving patient outcomes.

However, therapeutic enhancement of IL-10 activity carries inherent risks that require careful consideration. The anti-inflammatory signal dominance may create permissive environments for viral replication. The inhibition of the IL-10-IL-10R axis is being investigated for its ability to reduce viral persistence by counteracting immune suppression. Elevated serum IL-10 in chronic viral infections (e.g., HBV, HCV, HIV, SIV) is reversed by IL-10 signaling blockade during immunization, enhances antiviral T cell responses, and facilitates viral clearance [27,148,149]. Blocking IL-10/IL-10R signaling using in vivo anti-mouse IL-10R (clone 1B1.3A) was able to prevent lymphopenia, which contributes to enhancing the survival of mice infected with FMDV [9]. IL-10 inhibitors (e.g., IL-10-targeting phosphorodiamidate morpholino oligomers) that disrupt gene expression via aberrant splicing conferred resistance to Ebola virus infection [150,151]. In addition, the clinical potential of IL-10 blockade is further underscored by its prospective integration into combination therapies with antiviral agents, interferons, and other agents that neutralize critical components of T cell exhaustion, such as PD-1 [27]. These paradoxical effects highlight the need for spatiotemporal precision in IL-10 modulation strategies, even the balance point for using IL-10 treatment.

### 5.2. Future Research Challenges and Potential Developments in IL-10 Targeting Strategies

Despite the therapeutic potential of IL-10 modulation, significant challenges and knowledge gaps remain. This complexity needs a comprehensive understanding of IL-10’s role across various infections and patient populations. A primary challenge lies in the context-dependent duality of IL-10’s biological effects; while its anti-inflammatory properties may protect tissues in certain scenarios, excessive suppression of immune responses could paradoxically facilitate viral evasion and increase susceptibility to secondary infections. Thus, accurate disease models and advanced biomarker analyses are essential for anticipating and modulating the diverse effects of IL-10. The precise timing of IL-10/anti-IL-10 intervention remains a key challenge due to the lack of validated predictive biomarkers. Potential candidates—such as IL-10/TNF-α ratios reflecting anti-inflammatory effects, Treg kinetics relating to the immunosuppression, and co-inhibitory molecule expression (e.g., PD-1, TIM-3) on exhausted T cells—warrant further investigation to guide therapeutic strategies. Future advancements may arise from a deeper exploration of the molecular mechanisms governing IL-10’s interactions with immune cells and viral pathogens. Genetic and protein engineering approaches could yield IL-10 variants with tailored functions, enhancing therapeutic precision. Investigating the pathways that regulate IL-10 bioavailability and signaling may unveil new targets for more effective modulation of immune responses in viral infections. Incorporating computational biology and systems immunology into IL-10 research could facilitate breakthroughs by predicting complex interactions and guiding therapeutic design. By integrating these insights with high-throughput screening and personalized medicine approaches, the development of next-generation, patient-specific IL-10-based therapies may be realized, effectively addressing individual patient needs and the unique contexts of their diseases.

## 6. Conclusion and Perspectives

IL-10 is essential for limiting excessive inflammation and preventing tissue damage. However, its elevated levels can lead to significant immunosuppression, enabling viral persistence and contributing to chronic infections. The review underscores the effect of IL-10 as a crucial immunosuppressive cytokine under viral infection scenarios. The mechanisms through which IL-10 exerts immunosuppression are multifaceted, encompassing the reduction in pro-inflammatory cytokines, inhibition of antigen presentation, modulation of T cell activation, and induction of immune cell death and depletion of T cell function. IL-10 dampens the generation of crucial pro-inflammatory cytokines, thereby reducing recruitment and activation of effector immune cells necessary for effective viral clearance. Additionally, IL-10 hampers the antigen presentation process, further undermining the immune response. This duality of IL-10’s function underscores its potential attractiveness as a therapeutic target and a biomarker for viral infections.

Future research must focus on unraveling the situation-specific impacts of IL-10 in various viral infections and patient populations. For instance, in chronic HIV infection, elevated IL-10 drives immune exhaustion by impairing CD8+ T cell function, whereas in acute influenza infection, IL-10 deficiency exacerbates pulmonary immunopathology. Additionally, the parameters include exhaustion marker kinetics (PD-1, TIM-3), viral load dynamics, and tissue-specific immune profiling would be assessed. Modulation Approaches should be taken by time-dependent IL-10R blockade or rIL-10. Understanding the molecular pathways that regulate IL-10 signaling will be vital for developing targeted therapeutic strategies. Moreover, the potential for combining IL-10 modulation with existing antiviral therapies and immune checkpoint inhibitors (e.g., PD-1 blockade) presents an exciting avenue for enhancing treatment efficacy. This promising antiviral strategy carries risks of cytokine storms (especially in high viral load patients) and autoimmune exacerbations. Effective risk mitigation necessitates: (1) HLA-haplotype and autoantibody-based patient stratification; (2) pharmacodynamically optimized IL-10-primed sequential dosing coupled with real-time cytokine surveillance; and (3) prophylactic corticosteroids or spatially restricted IL-10 delivery systems. This multidimensional strategy enable safer clinical translation while maintain synergistic immune enhancement. This review emphasizes the significance of IL-10 in mediating immunosuppression during viral infections and its implications for therapeutic intervention. Continued investigation into IL-10’s immunomodulatory functions will pave the way for innovative approaches designed to enhance patient results in the treatment of viral illnesses.

## Data Availability

Not applicable.
